# Total Hip Arthroplasty Combined With Subtrochanteric Transverse Shortening Osteotomy: Factors Associated With Delayed Union at the Osteotomy Site

**DOI:** 10.5435/JAAOSGlobal-D-20-00056

**Published:** 2020-08-04

**Authors:** Toshiyuki Kawai, Koji Goto, Yutaka Kuroda, Shuichi Matsuda

**Affiliations:** From the Department of Orthopedic Surgery, Kyoto University Graduate School of Medicine, Sakyo-ku, Kyoto, Japan.

## Abstract

**Background:**

Total hip arthroplasty (THA) with subtrochanteric shortening osteotomy for Crowe type IV hips poses the risk of nonunion at the osteotomy site. The aim of this study was to analyze the factors that affect the bone union rate at the osteotomy site.

**Methods:**

We retrospectively reviewed a consecutive series of 27 THAs with subtrochanteric transverse shortening osteotomy performed for Crowe type IV hips. The effects of patient-related and surgery-related factors on the risk of delayed union were analyzed using univariate and multivariate regression analyses.

**Results:**

The mean follow-up period was 10.0 (1.4 to 19.1) years. The implant survival rate was 87.8% (95% confidence interval: 60.2% to 97.2%) at 10 years. The length of femoral bone resection was the only factor associated with the risk of delayed union. Longer bone resection lengths were significantly correlated with the reduced risk of delayed union (odds ratio: 0.63 [0.030 to 0.90], *P* = 0.0013). Other variables, including the use of a cement stem (*P* = 0.34) and the presence of a gap >1 mm at the osteotomy site (*P* = 0.98), were not associated with the risk of delayed union.

**Discussion:**

THA with subtrochanteric transverse osteotomy provides satisfactory long-term results for Crowe type IV hips. For shorter required femoral resection lengths, the risk of delayed union was higher. A longer resection could permit fabrication of longer autologous longitudinal bone struts and likely contributes to enhanced stability at the osteotomy site.

Crowe type IV developmental dysplasia of the hip is one of the most challenging types of hip deformity to reconstruct. Total hip arthroplasty (THA) for Crowe IV high hip dislocation often requires subtrochanteric shortening osteotomy to allow the acetabular implant to be located at the level of the anatomic hip center. Without the shortening osteotomy, limb lengthening would likely be more than 4 cm, which reportedly increases the risk for nerve palsy.^1-3^

Mid-term and long-term results of THA with subtrochanteric shortening osteotomy have been reported in several studies. However, most studies had a small number of patients, and various surgical techniques were used. Consequently, the results were not consistent. Implant survival rates with implant revision as the end point were 76% to 83% at 5 years.^4-6^ However, some reports showed more encouraging survival rates of 92% to 94% at 10 years.^7,8^

A major complication associated with subtrochanteric shortening osteotomy is nonunion at the osteotomy site. According to the literature, the nonunion rate of transverse osteotomy is 1.3% to 20%.^4,6,7,9,10,11,12,13,14,15,16^ Previous reports tended to focus on the various osteotomy techniques used to reduce the risk of nonunion or delayed union. However, to the best of our knowledge, no study has analyzed the factors affecting bone union at the osteotomy site.

The aims of the current study were to evaluate the long-term results of THA combined with subtrochanteric transverse shortening osteotomy for Crowe IV hip and to determine the factors that affect the risk of delayed union at the osteotomy site.

## Methods

This was a retrospective study of primary THA combined with subtrochanteric shortening osteotomy performed for Crowe type IV developmental hip dysplasia between May 2000 and January 2016. We performed 44 THAs in 35 patients for Crowe IV hips during this period. Of these, 28 hips (62.2%) in 21 patients (20 women and 1 man) required subtrochanteric shortening osteotomy. Some cases of this series were included in a previous report published in 2011.^12^ All patients provided informed consent, and the study protocol was approved by the institutional review board of our hospital.

The 44 hips under consideration were categorized into two subgroups: Crowe type IV-a and Crowe type IV-b. A joint was regarded as type IV-a if the formation of a false acetabulum was evident, whereas it was regarded as type IV-b if the dislocated femoral head was located within the abductor muscle mass. The incidence of shortening osteotomy in each type of hip was calculated and analyzed statistically. During THA, attempts were made even for Crowe IV hips to locate the acetabular implants at the anatomical center and to reduce the femoral head into the acetabulum without performing shortening osteotomy. If this approach seemed impossible without osteotomy because of tight soft tissue around the hip joint, the decision to perform osteotomy was made intraoperatively.

One patient was lost to follow-up after she moved to another city at 4 months after the index surgery. This meant that, in the current study, 27 hips in 20 patients underwent THA with subtrochanteric shortening osteotomy. For 25 of these 27 hips (92.6%), the index THA was the first hip surgery. One patient had previously undergone valgus osteotomy surgery, and the other patient had undergone Salter osteotomy in their childhood. Of the 25 complete dislocations, THA with subtrochanteric shortening osteotomy was performed on both hips in 6 patients and on unilateral hip in 13 patients. For the unilateral cases, the contralateral hip was categorized as Crowe type IV requiring THA without subtrochanteric shortening osteotomy in two hips, osteoarthritic change with Crowe type I subluxation in five hips, osteoarthritic change with Crowe type III subluxation in one hip, and normal in five hips.

During the same period (from May 2000 to January 2016), we performed 20 THAs for Crowe III hips, among which only one patient (5%) required subtrochanteric osteotomy. This case was not included in the current study. The mean patient age at the time of the index THA was 63.4 years (SD: 10.5, range: 42 to 85 years). The average follow-up period was 10.0 years (SD: 5.5, range: 1.4 to 19.1 years) after index surgery. The reason for THA in all patients was buttock pain and low back pain with or without knee pain resulting from the dislocated hip. The pain was associated with stiffness and limitation of activities.

Noncemented stems were used in 17 hips (S-ROM [DePuy Synthes] in 16 hips and Cannulock [Orthodesign] in 1 hip], and cemented stems were used in 10 hips (Kyocera type 6 [Kyocera] in 6 hips and H3 [Kyocera] in 4 hips: both were Charnley-type stems). A noncemented cup was used in one hip (AHFIX Q3 [Kyocera] cup with an outer diameter of 42 mm). Cemented acetabular components made of highly cross-linked polyethylene were used in 26 hips (CLHO in 14 hips, CP socket in 10 hips, and EXL GP socket in 2 hips, all supplied by Kyocera).

Clinical assessments were made using the Japanese Orthopedic Association (JOA) hip score, which allocates 40 points for pain, 20 points for range of movement, 20 points for walking ability, and 20 points for activities of daily living. The maximum total score is 100 points.

Limb lengthening was calculated by subtracting the length of the resected femur from the brought-down distance of the prominent point of the lesser trochanter.^17^ Whereas the length of the resected femur was measured intraoperatively, the brought-down distance was measured by comparison between postoperative and preoperative radiographs and was corrected using a calibration marker.

The location of the postoperative hip center was defined as the distance from the teardrop. The vertical height of the hip center was measured perpendicular to the interteardrop line. The horizontal distance of the hip center was measured along the line as the distance from the lowest point of the teardrop. The postoperative hip center was considered to be within the anatomical hip center if it met the following two criteria: (1) superior displacement of <15 mm from the approximate femoral head center^18^ and (2) superior displacement of <35 mm from the interteardrop line.^19^

**Figure 1:**
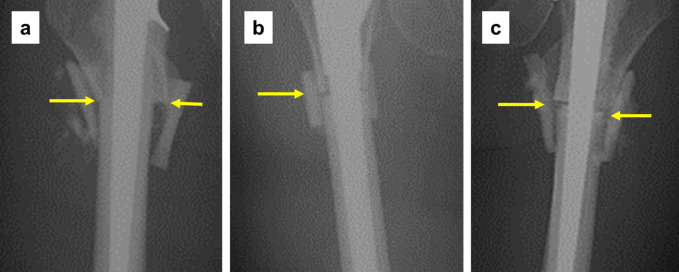
Radiographs showing three types of osteotomy site: **A,** without a noticeable gap, (**B**) with a gap of more than 1 mm, and (**C**) with cement interposition within the gap at the osteotomy site. Arrows indicate osteotomy sites.

The condition of the osteotomy site on radiographs immediately after surgery was classified into the following three types (Figure 1): (1) contact without a noticeable gap, (2) the presence of a gap >1 mm without apparent cement interposition, and (3) the presence of a gap >1 mm with cement interposition.^20^

Bone healing at the osteotomy site was evaluated using serial radiographs. The time to union, measured as months after the index surgery, was noted at the first radiographic evidence of healing of the osteotomy site. Postoperative radiographs were taken every 1 to 2 months until 6 months after surgery and thereafter every 2 to 3 months until bone union was confirmed. If bone union was not confirmed within 12 months after index surgery, the case was defined as delayed union. The length of the resected femoral bone was measured intraoperatively using a ruler.

### Surgical Procedures

THA was carried out using the direct lateral approach (Hardinge or Dall) with the patient in the lateral decubitus position. The pulvinar, which covers the inferior margin of the acetabulum, was identified to confirm the location of the original acetabulum. After reaming the cavity, a bone autograft obtained from the femoral head and neck was used to augment the roof of the undeveloped original acetabulum in all 27 procedures. The graft was fixed with at least two biodegradable screws and was then reshaped with a reamer so that the graft did not obstruct insertion of the socket. The femoral implant was implanted above the level of the lesser trochanter. The decision on whether subtrochanteric osteotomy should be performed was based on the intraoperative assessment of soft-tissue tension on the attempt to reduce the femoral head into the acetabular implant. Trial reduction was attempted with trial components in place. If reduction was impossible because of soft-tissue or neurovascular tension, the trial stem was removed, and subtrochanteric transverse osteotomy was performed below the lessor trochanter. With the trial stem in the proximal fragment, the stem head was reduced into the cup, and then, manual traction was applied to the distal femur by pulling the leg. The length of the bony overlap was measured, and this segment was resected from the distal femur by performing a transverse cut with a bone saw. For cemented stems, cementing was done using a third-generation cementing technique with both femoral fragments held with bone clamps. The osteotomy site was covered with longitudinally divided fragments of subtrochanteric bone that was resected during shortening osteotomy or with bone chips from the resected femoral fragment. When onlay grafts were used, they were fixed with metal wires or polyethylene cables. Patients were free to walk with two supports after 3 to 7 days. Full weight-bearing was usually allowed after 6 to 8 weeks.

### Statistical Analysis

Differences in proportions were calculated using the chi-square test. Differences in means were calculated using the Wilcoxon test to compare two groups. *P* values of <0.05 were considered significant. For univariate regression analysis, the dependent variable was the incidence of delayed union at the osteotomy site, and the independent variables were age, body mass index (BMI), the length of bone resection, the length of limb elongation, use of a cemented stem, the presence of a gap >1 mm at the osteotomy site, and the use of chip graft versus allogeneic bone plates at the osteotomy site. For multivariate logistic regression analysis, the dependent variable was the incidence of delayed union, and the independent variables were parameters reaching a significance level of *P* < 0.1 in the univariate analysis. The cumulative bone union rate at the osteotomy site and the component survival rate (with revision surgery for any reason as the end point) were evaluated using the Kaplan–Meier method with 95% confidence intervals (CI). All statistical analyses were performed using JMP Pro 14 software (SAS Institute).

## Results

Among the 26 type IV-a hips, only 11 (42.3%) required subtrochanteric shortening osteotomy. By contrast, 16 of 18 (88.9%) type IV-b hips required osteotomy. The difference was statistically significant (*P* = 0.0018) (Table 1). Demographic data for the patients and data on the 27 hips of patients who underwent THA with subtrochanteric shortening osteotomy are shown in Table 2. The average follow-up period for the whole series was 10.0 years (range: 1.4 to 19.1 years). The JOA hip score increased from a mean value of 53.1 (SD: 18.3, range: 23 to 79) preoperatively to 80.7 (SD: 7.8, range: 66 to 95) at the last follow-up. Functional hip score parameters at the last follow-up compared with the preoperative data are shown in Table 3. The JOA score improved in all hips. Postoperatively, dislocation occurred in two hips (7.4%). One patient, who was 70 years old at the time of the THA, experienced posterior hip dislocation when she crouched 2 weeks after the surgery. Manual closed reduction was performed, and the hip has not become dislocated again. The other patient, an 85-year-old woman, suffered posterior dislocation twice at 3 and 5 weeks postoperatively. On both occasions, she bent over to pick up something from the floor and experienced dislocation. Closed reduction was performed both times, and after the second reduction, she has not suffered further dislocation. No cases of infection or nerve palsy were observed.

**Table 1:** The Number of Patients Who Required Subtrochanteric Shortening Osteotomy Combined With Total Hip Arthroplasty

	Total	Without Osteotomy	Osteotomy Required	*P*
Type IV-a	26	15	11	0.0018
Type IV-b	18	2	16	

**Table 2:** Demographics of the Patients and Data on the 27 Hips of Patients Who Underwent THA With Subtrochanteric Shortening Osteotomy

	Overall	Union by 12 mo	Delayed	*P*
Age (year)	63.4 (SD: 10.5, 42-85)	64.4 (SD: 11.1)	60.0 (SD: 7.3)	0.20
Body mass index (kg/m^2^)	21.3 (SD: 3.6, 15.6-29.3)	21.2 (SD: 3.5, 15.6-29.3)	21.9 (SD: 4.0, 18.4-26.4)	0.47
Resection length (mm)	37.4 (SD: 12.9, 20-60)	40.4 (SD: 12.4)	24.4 (SD: 3.6)	0.0062
Leg elongation (mm)	26.8 (SD: 11.1, 0-45)	27.7 (SD: 10.8)	23.0 (SD: 12.6)	0.38
Cemented stem	10/27	7/22	3/5	0.24
Gap>1 mm at osteotomy site	10/27	9/22	1/5	0.38
Chip graft instead of bone plate	4/27	2/22	2/5	0.079

**Table 3:** Japanese Orthopaedic Association Score Before Surgery and at Final Follow-up

	Before Surgery	Final Follow-up
Pain	21.7 (SD: 13.7, 10-40)	36.8 (SD: 4.9, 20-40)
ROM	10.5 (SD: 3.6, 2-14)	13.4 (SD: 3.6, 8-20)
Walk	8.2 (SD: 4.1, 5-18)	14.4 (SD: 3.7, 10-20)
ADL	12.8 (SD: 2.8, 10-16)	16.2 (SD: 1.4, 12-20)
Total	53.1 (SD: 18.3, 23-79)	80.7 (SD: 7.8, 66-95)

ADL = activity of daily living, ROM = range of motion

The mean length of the excised femoral segment was 37.4 mm (SD: 12.9, range: 20 to 60 mm), and the mean leg elongation of the treated limb was 26.8 mm (SD: 11.1, range: 0 to 45 mm). In five cases, the leg elongation was more than 40 mm, but nerve palsy was not observed in any case.

The mean location of postoperative hip center was 24.0 mm (SD: 2.9, 16 to 29 mm) lateral from the teardrop along the interteardrop line and 18.9 mm (SD: 4.3, 10 to 26 mm) superior to the interteardrop line. These results indicated that all the acetabular components were within the region of the anatomical hip center: i.e., they had a superior displacement of <15 mm from the approximate femoral head center^18^ and a superior displacement of <35 mm from the interteardrop line.^19^

Bone union at the osteotomy site was evident by 12 months after surgery in 22 of 27 hips (81.5%). Among the 5 hips (18.5%) in which the osteotomy site had not healed within 12 months, 1 hip underwent stem revision surgery using allogeneic strut bone plates at 3 years and 1 month after the THA. Bone healing was confirmed after the revision surgery. The course of this particular patient was previously reported.^12^ All the five hips that did not heal by 12 months were followed up more than 18 months.

**Figure 2:**
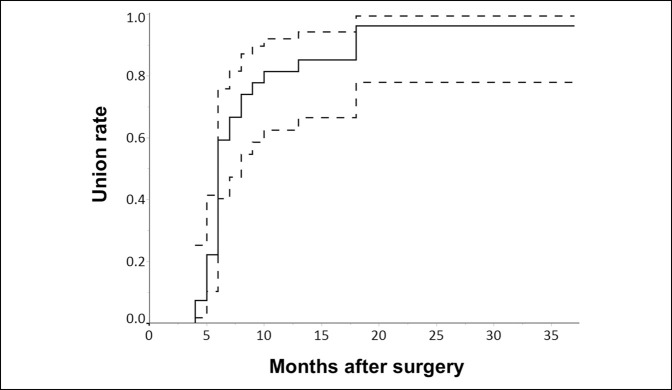
Chat showing the cumulative bone union rate at the osteotomy site. The dashed lines indicate the 95% confidence interval.

The cumulative bone union rate after the index surgery is shown in Figure 2. The union rate was 59.3% (95% CI: 40.3% to 75.8%) at 6 months, 81.5% (95% CI: 66.5% to 94.3%) at 12 months, and 96.3% (95% CI: 77.9% to 99.5%) at 18 months.

In total, 17 osteotomy sites were type 1 (without a gap), and 4 (23.5%) of these experienced delayed union. Of the eight type 2 osteotomy sites (gap >1 mm), only one (12.5%) experienced delayed union. There were only two type 3 osteotomy sites (gap >1 mm with cement interposition), both of which achieved bony union at 6 months after surgery. Although the delayed union rate tended to be lower when there was a gap larger than 1 mm at the osteotomy site (10% vs 23.5%), the difference was not statistically significant (*P* = 0.38).

**Figure 3:**
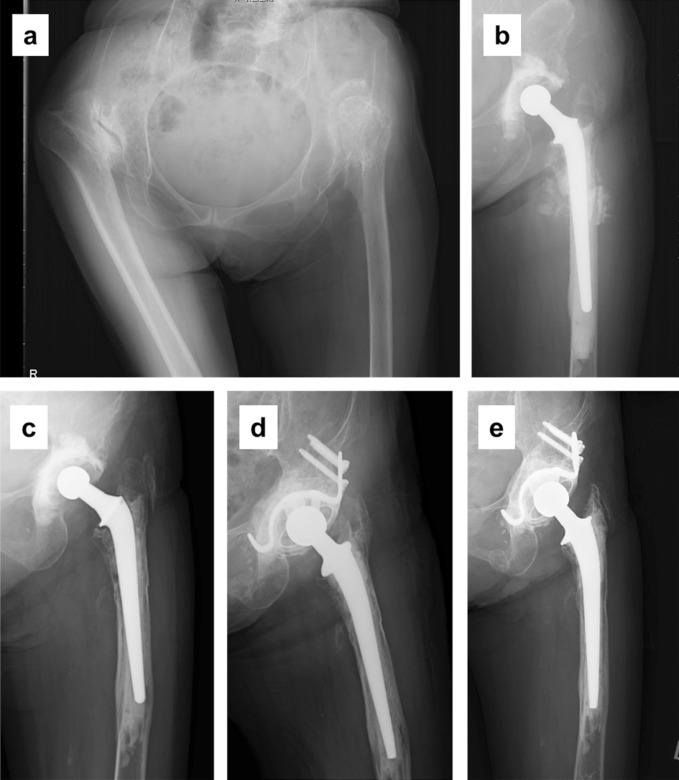
Radiographs of a 69-year-old woman who underwent THA combined with subtrochanteric shortening osteotomy for Crowe IV hip. **A,** Before surgery and (**B**) just after cemented THA with osteotomy. **C,** Apparent stem loosening at 9.5 years after surgery. **D,** Just after revision surgery with Kyocera type 6 cemented stem. **E,** No loosening was observed at 9 years after revision surgery. THA = total hip arthroplasty

Two patients underwent revision surgery. One aforementioned patient underwent stem revision combined with allografting due to nonunion at the osteotomy site at 3 years and 1 month after index THA. The other patient underwent revision surgery due to aseptic stem loosening at 9 years and 6 months after index THA (Figure 3). The acetabular implant did not show signs of loosening in plain radiographs, but it was exchanged simultaneously when the 22-mm head used for the index surgery was replaced with a 26-mm head.

**Figure 4:**
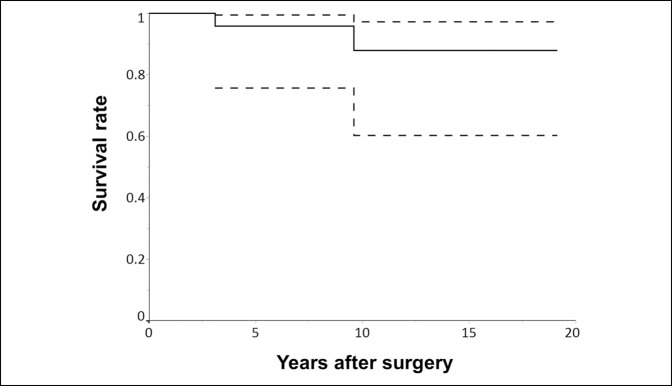
Chart showing the implant survival rate with implant revision for any reason as the end point. The dashed lines indicate the 95% confidence interval.

The implant survival rate (with implant revision for any reason as the end point) was 95.8% (95% CI: 75.6% to 99.4%) at 5 years, 87.8% (95% CI: 60.2% to 97.2%) at 10 years, and 87.8% (95% CI: 60.2% to 97.2%) at 15 years (Figure 4).

The results of univariate regression analysis are shown in Table 2. The following factors were not associated with the incidence of delayed union at the osteotomy site: age, BMI, the length of limb elongation, the use of a cemented stem, the presence of a gap >1 mm at the osteotomy site, and the use of chip graft versus an allogeneic bone plate at the osteotomy site. The length of bone resection was statistically significantly correlated with the incidence of delayed union (*P* = 0.0062); larger bone resection lengths were associated with a lower risk of delayed union.

Multivariate logistic regression analysis was performed with age, BMI, the length of limb elongation, the use of a cemented stem, the presence of a gap >1 mm at the osteotomy site, and the use of chip graft versus an allogeneic bone plate at the osteotomy site as dependent variables and the occurrence of delayed union as the independent variable. The length of the resected femur was the only significant risk factor for delayed union (OR: 0.63 [95% CI: 0.030 to 0.90], *P* = 0.0013), indicating that when the bone resection length was larger, the risk of delayed union was lower (Table 4).

**Table 4:** Odds Ratios and *P* Values Obtained for Each Variable by Multivariate Logistic Regression Analysis

	Odds Ratio (95% CI)	*P*
Age (yr)	1.03 (0.73-4.82)	0.90
BMI (kg/m^2^)	1.27 (0.57-15.23)	0.53
Resection length (mm)	0.63 (0.030-0.90)	0.0013
Leg elongation (mm)	1.11 (0.87-4.05)	0.47
Cemented stem	146.96 (0.0049-4362097.10)	0.34
Gap>1 mm at osteotomy site	0.94 (0.0073-121.98)	0.98
Chip graft instead of bone plate	5.54 (0.0013-22417.85)	0.69

**Figure 5:**
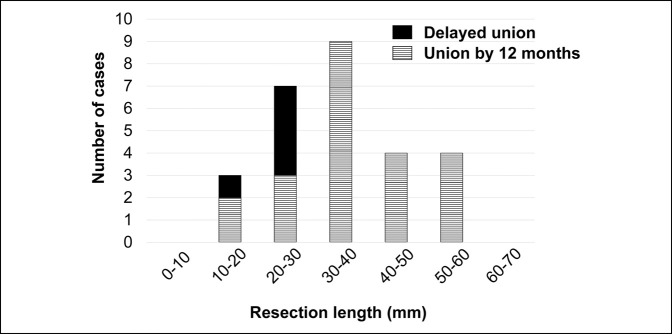
Chart showing the number of cases with delayed union and successful union by 12 months after surgery according to the femoral resection length.

The distribution of cases with delayed union and with successful union by 12 months after surgery according to the length of resection is shown in Figure 5. Of 10 cases with resection lengths of less than 30 mm, five had union by 12 months, whereas all 17 cases with resection lengths of 30 mm or more had bone union by 12 months. Moreover, this difference was statistically significant (*P* = 0.0012).

## Discussion

Although THA for Crowe type IV developmental hip dysplasia is an effective procedure to improve hip function, the procedure poses various difficulties and carries the risk of serious complications. For mechanical and anatomic reasons, the best location to place the socket is at the level of the true acetabulum. In the present study, acetabular components were placed at the level of the true acetabuli according to the criteria given in the literature.^18,19^

One of the most serious risks associated with THA for Crowe type IV hip dislocation is nerve palsy. If femoral subtrochanteric shortening osteotomy is chosen to avoid this risk, there is the additional risk of nonunion of the osteotomy site, which reportedly occurs in 1.3% to 20% of cases.^4,6,7,9,10,11,12,13,14,15,16^ Several research groups have attempted to minimize the risk of delayed union or nonunion by using various types of osteotomy techniques, e.g., oblique,^21^ double-chevron,^22^ and complex step-cut.^23,24^ However, to the best of our knowledge, no study has analyzed the factors associated with the risk of delayed union.

The main finding of the current study was that when the femoral resection length was larger, the incidence of delayed union was lower. The resected femoral fragments were longitudinally divided into two or three pieces and were fixed at the osteotomy site as autologous bone plates. We assume that larger bone grafts will provide more stable fixation and a larger contact area between the femoral shaft and the bone graft, which could lead to faster indirect healing between the proximal and distal fragments via bone graft. However, the length of femur to be resected should be based on the distance between the femoral head and the acetabulum component when the reduction force is fully applied.

That bone resections of more than 30 mm provided a better union rate does not mean that surgeons should resect more than 30 mm in all cases. Excessive resection decreases the muscle tension and poses the risk of postoperative dislocation. In our opinion, if the required resection length on trial reduction is more than 30 mm, the resection length should be that required length. That would provide sufficient bone grafts for the osteotomy site and retain adequate muscle tension. If the required bone resection is 15 to 30 mm, the resection length should again be the required length. To decrease the risk of delayed union in these cases, rather than increasing the resection length, the use of additional stabilizers such as a short metal plate should be considered. When the necessary resection length seems to be less than 15 mm to reduce the femoral head into the acetabular implant, we advise against performing subtrochanteric shortening osteotomy. In such cases, we recommend that surgeons place the stem deeper by cutting the base of the femoral neck to the level of the lessor trochanter using a previously described method.^17^

It remains to be clarified how often subtrochanteric osteotomy is needed in THAs for Crowe IV hips. Only a few reports have mentioned the frequency of Crowe IV cases that require subtrochanteric shortening osteotomy with respect to all Crowe IV cases encountered during the same study period, with the proportion ranging from 15.8% to 62%.^17,25,26^ In the current study, 61.4% of Crowe IV hips required subtrochanteric shortening osteotomy. Only 11 of 26 Crowe IV-a hips (42.3%) required subtrochanteric shortening osteotomy to place the hip center at the level of the anatomical hip center, whereas 16 of 18 Crowe IV-b (88.9%) hips underwent osteotomy. More than half of Crowe IV-a hips did not require shortening osteotomy to locate the acetabular implant at the level of the anatomical acetabulum and to reduce the femoral head into it.

The use of cement for the femoral implant was not correlated with delayed union in the current study. Many studies have reported union rates for subtrochanteric shortening osteotomy, and there have been no obvious differences in the incidence of delayed union between cemented stems and noncemented stems. In the current study, 3 of 10 cases exhibited delayed union in the cemented stem group, whereas 2 of 17 cases showed delayed union in the noncemented stem group. This difference was not statistically significant (*P* = 0.34 by the multivariate model). Currently, we use a standard sized cemented stem with a rectangular cross section for THA combined with subtrochanteric shortening osteotomy. The application of a stem with a rectangular cross section should provide better rotational stability than a cemented stem with a cylindrical geometry.

In the current study, a gap of around 1 mm at the osteotomy site was seen in 10 hips. In 9 of these 10 hips, bone union was confirmed by 12 months after the index surgery. Cement interposition within the gap was observed in two hips. Both of these hips showed bone healing by 6 months. A gap of around 1 mm at the osteotomy site was not found to be necessarily detrimental, and in these cases, bony union involving the onlay grafts can be expected.

Although older age has been shown to negatively affect the processes of bone healing,^27^ patient age at the time of surgery was not associated with a risk of delayed union in the current study. The amount of limb elongation was also not correlated with the incidence of delayed union in this study. When limb elongation is large, the muscle tension that pulls the distal femoral fragment proximally would be higher. However, that mechanism apparently did not significantly affect the timing of bone healing.

The limitations of the current study include the retrospective nature of the analysis and the heterogeneity of the cohort. In addition, 27 cases were involved in the present study, in which delayed union was seen in only 5 cases. This small number also represents a limitation of the model in this study. Another limitation would be that judging union is subjective based on plane radiographs, not on three-dimensional images. There would be a possibility that some of delayed union that united at 18 months were already in the middle of uniting at 12 months. To draw a definite conclusion on the correlation of the resection length and the stability at the osteotomy site, biomechanical studies should be conducted in the future.

In conclusion, THA with subtrochanteric transverse shortening osteotomy provided an encouraging long-term implant survival rate of 87.8% (95% CI: 60.2% to 97.2%) at 10 years and 87.8% (95% CI: 60.2% to 97.2%) at 15 years. When the femoral resection length was smaller, the risk of delayed union was higher. Surgeons may need to consider additional stabilization at the osteotomy site if the required resection is less than 30 mm and adequate strut bone grafting at the osteotomy site could not be achieved.
